# Real-world evidence for primary care: A primer on observational research

**DOI:** 10.4102/phcfm.v17i2.5197

**Published:** 2025-11-25

**Authors:** Klaus B. von Pressentin, Keneilwe Motlhatlhedi, Malo Musende, Tibor Schuster

**Affiliations:** 1 Division of Family Medicine, Department of Family, Community and Emergency Care (FaCE), Faculty of Health Sciences, University of Cape Town, Cape Town, South Africa; 2 Department of Family Medicine and Public Health, Faculty of Medicine, University of Botswana, Gaborone, Botswana; 3 Department of Epidemiology, Biostatistics and Occupational Health, McGill University, Montreal, Canada; 4 Department of Family Medicine, McGill University, Montreal, Canada

**Keywords:** primary health care, primary care, observational study, cohort studies, case–control studies, cross-sectional studies, causal inference, health services research

## Abstract

Observational studies offer a non-experimental and minimally disruptive approach for generating real-world evidence, making them particularly valuable for informing clinical practice, research and health system strengthening – especially in primary care. This article, part of the *African Journal of Primary Health Care & Family Medicine* (PHCFM) methods series, introduces key observational study designs including cross-sectional, cohort and (nested) case–control studies and discusses their application in doctoral-level research. Drawing on historical and contemporary examples, we examine methodological considerations, ethical issues and modern analytical strategies essential for the careful planning and execution of observational research. By integrating conceptual frameworks and causal inference methods, this primer aims to equip researchers at different career stages with a foundational understanding of how to choose and implement observational designs that are both methodologically robust and relevant to primary care contexts.

## Introduction

Observational studies have long been pivotal in medical discovery, from Jenner’s^1^ recognition of smallpox immunity to Nightingale’s^2^ pioneering hospital reforms. In modern evidence-based primary care, they remain essential for evaluating interventions, understanding disease patterns and informing practice guidelines and policies.

While there is a need for robust evidence generated through randomised controlled trials (RCTs), rigorously conducted observational studies continue to offer indispensable insights that are often unattainable through experimental designs alone.^3,4^

This article is part of a series intended to guide doctoral researchers and supervisors in methodological approaches. Readers are encouraged to consult companion articles in this series to inform the most suitable designs required to answer the overarching question of their doctoral study.^5,6^

In this article, key takeaways for the reader are flagged with the ✍🏻 symbol and emphasised in *italicised active tense*.

## Real-world evidence: Balancing experimental versus observational approaches

Both experimental and observational studies play a crucial role in advancing evidence-based medicine, offering distinct yet complementary insights that inform clinical decision-making and the development of effective interventions. Among experimental designs, RCTs are considered the gold standard for establishing and quantifying causal relationships. However, the strong internal validity of RCTs often comes at the expense of generalisability, as RCTs frequently exclude key populations and may therefore fail to reflect the realities of routine clinical practice.

Observational studies, in contrast, excel at capturing the complexities of the real world. They are designed to mirror everyday clinical settings, often embracing broad inclusion criteria and large, diverse populations. These studies are particularly valuable for evaluating long-term outcomes, ongoing interventions and broader questions of health service delivery and system performance. In observational studies, exposure is not assigned by the researcher, making these designs well-suited for investigating exposures that cannot be ethically randomised, such as lifestyle factors, environmental risk factors and social determinants of health (SDH). This greater real-world relevance, however, comes with a trade-off: observational studies are vulnerable to *confounding bias*, meaning that associations between exposures and outcomes may be explained partly or entirely by other factors, namely, exogenous variables not appropriately accounted for. Moreover, data obtained in non-experimental settings, even if derived from (electronic) health records or administrative databases, can vary in quality. Missing covariates, data entry errors, undocumented changes in clinical practice and limited oversight can further undermine the strength of evidence generated by observational research, chiefly through mechanisms referred to as *selection bias*^7^ and *measurement* error.^8,9^

In response to these challenges, modern causal inference paradigms enable researchers to articulate, assess and fulfil the conditions necessary for robust inferences from observational data, thereby strengthening their relevance for guiding effective interventions.^10^ In essence, these frameworks enable researchers to approximate counterfactual comparisons that closely mimic the conditions of RCTs, accounting not only for confounding but also for other complex issues such as selection bias.^11^

✍🏻 *Observational study designs, guided by modern causal inference frameworks, approximate the conditions of randomised trials while retaining the unique advantages of real-world generalisability and practice relevance. In this way, observational study designs complement experimental approaches in building a comprehensive understanding of the determinants of health across multiple levels within the primary care ecosystem*.

## Before the research begins

Planning for observational research should commence early in the conceptual development of a doctoral project. The timeline for protocol development, recruitment or sampling and data availability and acquisition across various observational designs should be considered.

Before embarking on any meaningful research project, a crucial first step is to establish a clear and focused research question. This foundational element serves as the ignition point that drives the entire study’s design, execution and, ultimately, the interpretation of the study findings. Observational studies are no exception, as they can vary widely in purpose, scope and methodology – all depending on the knowledge the investigators are aiming to generate from the research.

In observational studies, theoretical and conceptual frameworks can help guide the formulation of the research question, the selection of variables and the interpretation of findings. For instance, a researcher investigating the relationship between socioeconomic status (SES) and the incidence of cardiovascular disease might use the SDH framework.^12^ This framework posits that health outcomes are shaped by social, economic and environmental factors, influencing both exposure patterns (e.g., diet, physical activity, access to care) and susceptibility to disease. By grounding the study in the SDH framework, the researcher can identify relevant covariates for measurement, hypothesise potential causal pathways and interpret associations in the context of broader structural influences, thus drawing a conceptual framework for the study. Other examples of commonly applied frameworks in observational research include Andersen’s Behavioural Model of Health Services Use (for studies on healthcare utilisation), the Health Belief Model (for behavioural and preventive health studies), Life Course Theory (for studies linking early-life exposures to later health outcomes) and Ecosocial Theory (for multilevel analyses of health disparities).^13^

Much like RCTs, observational studies benefit from a structured approach to framing research questions. One widely used framework to guide this process is patient or population, exposure, comparator and outcome (PECO); this is the PICO framework (commonly used to develop research questions for RCTs) adjusted to include exposure instead of intervention (see Box 1).

BOX 1 Framework to structure the research question.

**Components of the PECO framework**

**Population:** Who specifically is being studied? Defining the *target population* involves specifying well-defined inclusion and exclusion criteria. For example, one may intend to study adults with a particular condition, children in a specific region or healthcare workers in a particular setting. A clearly defined study population is essential for ensuring the generalisability of findings; however, it must also be balanced with the feasibility of obtaining relevant data for the defined population. For example, investigating the effect of understaffing on hospital discharge times may be impossible to answer using an observational study approach if no reliable data on health workers’ shift-uptake and/or discharge episodes are accessible through hospital records.

**Intervention/Exposure:** Unlike RCTs, where ‘intervention’ often implies a treatment or procedure under experimental control, in observational studies, this refers to the exposure or factor of interest. This could be an environmental exposure, a lifestyle factor, a medication or any other characteristic that is hypothesised to be either associated with or, under more stringent assumptions, even causally linked to a specific variable or factor of interest, that is, the outcome. This latter distinction is critical as it determines the methodological approach in study design and analysis, as well as the interpretation and implications of findings once the study has been completed.

**Comparator:** Observational studies often compare groups with and without the exposure or between different levels of exposure. Identifying appropriate comparators helps in assessing the association and, again, under certain conditions, even the causal relationship between exposures and outcomes by providing a well-defined reference group.

**Outcome:** What are the factors or variables to be measured in relation to the exposure? Typically, a single primary outcome must be defined to ensure the proper design of the study. Examples include health indicators such as disease incidence or measures related to health services. The pre-determination of one primary outcome is vital as every investigation of a research question offers opportunities for chance findings, that is, for results that emerge as a random artefact. With the increasing number of exposure–outcome relationships being examined, the likelihood of generating spurious findings that cannot be reproduced steadily increases. The probability of obtaining such false positive results is referred to as a Type I error and must be controlled for.

RCT, randomised controlled trials; PICO, population, intervention or exposure, comparator and outcome.

### Formulating research objectives

Research objectives serve as the concrete, operational steps that translate a study’s research questions into specific actionable components. They define how the research question will be addressed by outlining measurable targets, such as specifying the underlying quantity of interest (i.e. the estimand) and clarifying the interpretability of the respective estimated effect measures.

✍🏻 *In observational studies, research objectives should distinguish between measures of association answering the question ‘What is the apparent relationship between exposure and outcome?’ and counterfactual quantities (i.e., causal estimands) such as expected population-level treatment effects, which under more stringent assumptions aim to answer ‘What would happen if the exposure were modified for all or some members of the population?’ This distinction guides the selection of analytic methods and informs the practical implications of the findings*.

For example, age is strongly associated with the likelihood of a successful pregnancy, and this relationship can be quantified. However, age itself is not a modifiable exposure but rather a proxy for underlying biological factors that affect fertility. In a counterfactual sense, ‘increasing’ a woman’s age while holding all other attributes constant would not directly alter pregnancy success; the observed association reflects the influence of underlying conditions that correlate with age.

Similarly, SES is associated with the incidence of chronic conditions such as hypertension or diabetes, and this relationship can be quantified in observational studies. However, SES itself is not a directly modifiable exposure but a proxy for underlying determinants such as access to care, nutrition, housing stability or chronic stress. In a counterfactual sense, ‘increasing’ a patient’s SES without altering these underlying conditions would not necessarily change their risk of developing hypertension; the observed association reflects the influence of these mediating factors rather than SES per se, and it is difficult to conceive of a feasible, scalable intervention that could directly shift SES.

✍🏻 *These examples illustrate the principle of ‘no causation without manipulation’: exposures such as age or SES can be associated with outcomes, but because they are not directly manipulable, they cannot define clear causal effects on their own*.^15,16^ *To draw practice-relevant conclusions, causal inference relies on the consistency assumption*^17^ *– the requirement that the effect of an exposure or intervention is well defined and unambiguous when being applied consistently across individuals. At the same time, it is important to acknowledge that non-manipulable characteristics – such as race, age or broader socioeconomic position – can still be meaningful objects of study. While they may not correspond to direct interventions, careful analysis of their associations can reveal structural disparities, illuminate upstream mechanisms and inform which modifiable factors or policies might reduce inequities. In this sense, even non-manipulable factors contribute to a higher-level understanding of health determinants and can guide the identification of intervention points*.^18^

## Methodological considerations

Beyond formulating a well-grounded and answerable research question, a robust methodological approach is required to guide the research process. In observational studies specifically, it is key to integrate methodologies that address threats to the internal validity of findings – biases that experimental designs typically minimise through randomised exposure assignment.

### Guidance through planning and conducting observational research

The identification and successful integration of relevant methodology rely heavily on the expertise and support of the supervisory team. Supervisors guide doctoral students through the conceptual, ethical and practical dimensions of their research. Additionally, collaborating early with methodology experts and biostatisticians is critical. Their input on study design, data management and analytical strategies – especially when dealing with complex datasets or advanced techniques – enhances the scientific rigour and credibility of the research findings.

Throughout the research process, it is important to prioritise ethical approval, data governance and transparency in reporting. Adhering to established best practices and relevant guidelines, such as the Strengthening the Reporting of Observational Studies in Epidemiology (STROBE) and the Consensus Reporting Items for Studies in Primary Care (CRISP) guidelines, ensures that the research study meets high standards of quality and integrity.^19,20^

### Choosing an appropriate study design

**FIGURE 1:**
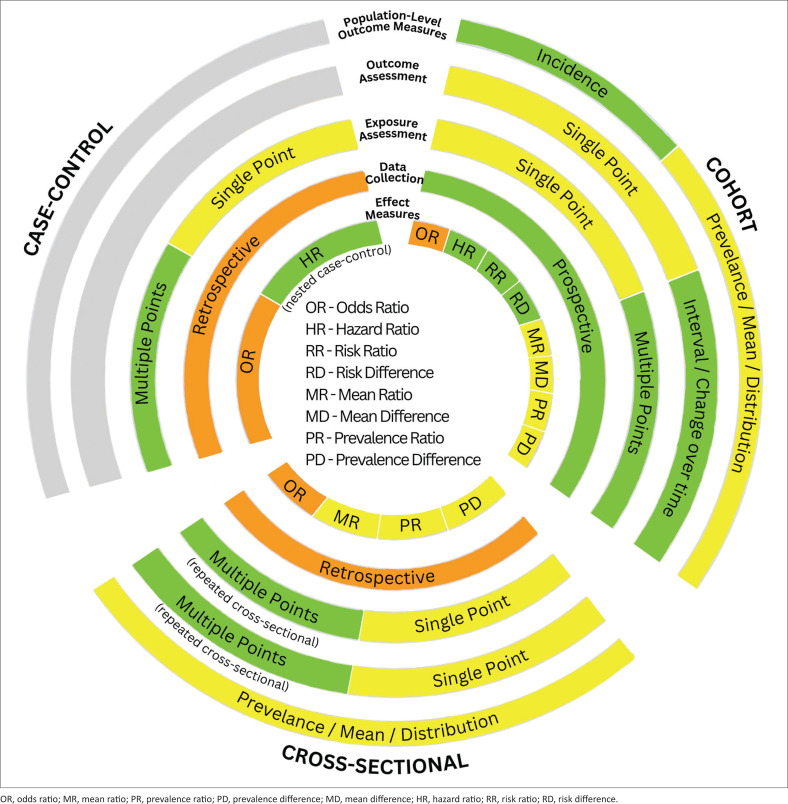
Observational study designs and outcomes – A Circos visualisation.

As discussed in the opening sections of this article, observational research plays a central role in generating practice-relevant evidence. It is not a single approach but a family of study designs, each with distinct strengths, limitations and applications. For doctoral students, recognising these differences is essential for selecting a design that fits the research question and generates evidence relevant to practice. The following subsections, along with the Circos plot in Figure 1, present three major observational designs: cohort, (nested) case–control and cross-sectional studies. Each track in the Circos plot represents a distinct design feature, including assessments of outcomes and exposures as well as effect measures.

The colours represent different levels of preference according to the strength of evidence generated and/or the interpretability of findings, with green indicating the most preferred option, followed by yellow and then orange.

#### Cross-sectional studies

Cross-sectional studies characterise covariate, exposure and outcome distributions within populations, help identify vulnerable subgroups and unmet health needs and provide preliminary evidence for exposure-outcome associations and potential underlying causes. A key design feature is that exposures and outcomes are measured simultaneously, typically at a specific index date or within a short calendar period; hence the name ‘cross-sectional’.

Cross-sectional studies are cost and time efficient and well suited for estimating prevalence, as data are collected at a single point in time. Common data sources include administrative databases, surveys and clinical records. However, because exposures and outcomes are measured simultaneously, it is often difficult to establish temporality or assess dose–response relationships, which limits causal interpretation. For example, if a researcher wished to examine whether prescribing antidepressants is associated with weight gain using patient health records, a single entry noting both weight and prescription status would not reveal which came first, leading to temporal ambiguity.

Another limitation of cross-sectional studies is the inability to assess data consistency over time, for example, in the presence of seasonal variation. When comparing subpopulations, outcome differences may be driven by variation in underlying determinants of health rather than by the exposure itself, making it essential to adjust for such confounding influences. Moreover, while cross-sectional studies enable the estimation of prevalence, they cannot provide incidence rates (i.e. the number of new cases arising in an at-risk population over time). One way to mitigate some of these limitations is through repeated cross-sectional studies, which allow researchers to track changes in exposures and outcomes across different time points and populations. This approach relies on key assumptions: consistent measurement over time, comparable sampling of populations and stable contextual factors that enable the results to be interpreted as temporal trends rather than methodological artefacts.

Although cross-sectional studies are often viewed as having limited inferential strength among observational designs, they can – under certain conditions – provide meaningful insights into cause–effect relationships. Modern causal diagrams help clarify these conditions, specifically when measurement error is minimal and the outcome under study is non-absorbing (i.e. not subject to selection bias because of its occurrence).^21^ For example, the determinants of acute conditions such as skin rash may be appropriately investigated using a simple cross-sectional design.

#### Case–control studies

Case–control studies allow the examination of multiple possible risk factors, such as lifestyle, genetics or environmental exposures, in relation to a single outcome. These studies begin by identifying individuals with the outcome of interest (cases) and selecting controls who do not exhibit this outcome. The major strength of case–control studies lies in their efficiency for studying rare or long-latency conditions without the need for large or lengthy cohort follow-up. Ideally, cases and controls come from the same source population, ensuring that controls represent the exposure distribution that would have been observed among cases had they not developed the outcome. Exposure history is then collected retrospectively to compare past exposures between groups, thereby assessing how odds of the outcome differ for exposed versus unexposed individuals. For example, a case–control study of Type II diabetes might recruit adults diagnosed with the condition as cases and age- and sex-matched adults without diabetes as controls. Researchers could then retrospectively assess multiple lifestyle factors such as past dietary patterns, levels of physical activity, smoking history and alcohol consumption to determine how these exposures differ between the two groups and whether they are associated with increased odds of developing diabetes.

A distinct variant, the nested case–control study, selects both cases and controls from within an established cohort. This design retains the efficiency advantages of case–control studies while reducing selection bias, as controls are drawn from a well-defined risk set at the time each case occurs. Nested studies also benefit from prospectively collected exposure data within the cohort, thereby mitigating recall bias and strengthening temporal inference.

✍🏻 *Case–control studies face several limitations. Because cases are oversampled relative to the base population, case–control studies cannot be used to estimate the prevalence or incidence rates of an outcome. As a result, the effect measure is limited to odds ratios, which may substantially overestimate risk ratios when the outcome is common (prevalence > 10%). This limitation is mitigated in nested case–control studies, as the Mantel–Haenszel odds ratio, when estimated within risk sets, provides a close approximation to the hazard ratio*.^22^

In case–control studies, valid inference depends on careful selection of controls to avoid exposure bias and on accurate assessment of exposure status. Misclassification, particularly because of recall bias, can compromise validity if exposures are self-reported; the use of health records can mitigate this risk although inaccuracies in diagnostic or administrative data can still lead to misclassification. Finally, outcomes must be clearly defined and reflect clinical relevance, as inconsistent or overly narrow definitions risk obscuring important associations.

#### Cohort studies

Cohort studies provide the most direct observational approach for examining how factors are associated with incident outcomes over time. Under stronger assumptions, and with appropriate covariate measurement and proper analytical approaches, they can provide robust evidence on whether exposures contribute to the development of those outcomes. Their defining feature is the grouping of individuals, initially free of the outcome of interest, into exposure-related cohorts that are then virtually followed to evaluate outcome occurrence. This temporal ordering supports causal interpretation and facilitates the estimation of incidence rates, which represent the frequency of new cases occurring in an at-risk population within a specified time period. Because they involve repeated observation of the same individuals over time, cohort studies are often also described as longitudinal studies, although not all longitudinal designs (such as repeated cross-sectional studies, which may involve changing populations) qualify as cohort studies.

Cohort designs can be prospective or retrospective (historical). In prospective studies, exposures and covariates are measured at baseline and updated during follow-up, giving oversight of time-varying factors that may contribute to the development of outcomes. The main limitation of prospective cohort designs is that they are resource intensive and require lengthy follow-up periods, often resulting in losses to follow-up that can introduce selection bias. Retrospective cohorts, by contrast, rely on existing databases or records to reconstruct exposure and outcome histories. They are more efficient in terms of time and cost, but their validity depends on the quality and completeness of the underlying data, as well as whether important covariates that act as confounding variables have been accurately recorded.

✍🏻 *A major strength of cohort studies is their ability to examine multiple outcomes for a single exposure, as well as to assess dose–response relationships and temporal patterns. This flexibility makes them specifically relevant in primary care, where clinical parameters, medication use or lifestyle factors may be considered exposures of interest in relation to diverse outcomes, whether investigated for associations or potential determinants of health*.

For example, an observational cohort study may stratify individuals based on the blood pressure treatment they receive as part of practice-based usual care, often reflecting prescriber preference. These exposure-defined cohorts can then be followed over time to estimate incidence rates within each group and to compare them across groups using hazard ratios, for outcomes such as cardiovascular disease, kidney disease, stroke and health service utilisation. Hazard ratios, however, are typically associational in nature and come with interpretational pitfalls – sometimes described as the ‘hazard of hazards’ – as they may not reflect causal contrasts even under proper adjustment.^23^ With appropriate causal modelling that targets average (marginal) treatment effects, cohort studies can move beyond associational hazards and yield more policy-relevant causal estimates.

## Common threats to the validity of findings generated from observational studies

In all observational study designs, addressing various sources of bias is critical for producing valid and credible results. A central challenge is confounding bias, which occurs when extraneous variables influence both the exposure and the outcome, potentially distorting the observed association. Controlling for confounding must occur at multiple stages: at the design stage, strategies such as careful matching or thoughtful control selection (in case–control studies) help balance confounders between groups. In cohort studies, it is essential to identify and measure all relevant factors that determine both exposure and outcome levels so they can be adjusted for during analysis. Among the tools available, propensity score methods serve as a powerful means to adjust for confounding by summarising the probability of exposure given measured covariates. Complementing these, directed acyclic graphs (DAGs) offer a conceptual framework for visualising causal relationships and identifying potential confounders, mediators and colliders, thereby guiding appropriate variable control. Although DAGs and propensity scores have gained prominence recently and are effective in addressing many longstanding challenges, they represent only part of a broader methodological ‘toolbox’ that researchers should explore through further reading of dedicated literature.

Another key concern is selection bias, which arises when the sampling process, sample frame or mechanisms for missing data influence the representativeness of the analytic sample. Propensity scores can also help mitigate selection bias by weighting or adjusting for factors related to sample selection. Directed acyclic graphs aid in detecting subtle selection biases, particularly by identifying collider stratification – a phenomenon where conditioning on a variable influenced by two other variables can induce spurious associations. This is typically visualised in DAGs as two arrows pointing into one node, signalling the need for caution.

### Sample size considerations

Lastly, to ensure that study findings are both precise and generalisable, researchers must carefully consider sample size and statistical power during the planning stage of their study. Adequate power ensures that the study can detect meaningful exposure effects with estimates that are precise enough to minimise the role of random error in interpretation. In practice, sample size calculations are guided by four key elements: the expected or minimally important effect size, the desired level of statistical power (one minus Type II error), the acceptable level of Type I error (i.e. statistical significance threshold) and the variability of the outcome (for discrete/continuous measures) or its incidence/prevalence (for binary outcomes). Together, these parameters determine whether a study is sufficiently equipped to yield results that are both reliable and interpretable. Analogous principles apply in Bayesian observational designs, where sample size is determined to ensure posterior estimates are sufficiently precise (e.g. narrow credible intervals) and that the probability of exceeding clinically meaningful thresholds is high enough to support robust decision-making.

## Integrating observational designs into doctoral protocols

Incorporating observational studies into a doctoral research project requires careful alignment between the research questions, study design and the practical realities of data availability and feasibility. Observational designs – whether cross-sectional, case–control or cohort – can function as standalone studies or be integrated within broader mixed-methods or sequential designs. For instance, a cross-sectional survey may identify patterns or generate hypotheses that can be later explored through qualitative interviews or longitudinal follow-up. The nature of the research question should guide the design selection, the theoretical framework underpinning the study and the methodological orientation of the doctoral candidate. Finally, all elements of planning, study design, implementation and analysis should be systematically specified and documented in a comprehensive research protocol.^24^

## Future directions

Observational studies are essential tools for doctoral researchers in primary care. When carefully designed and reported, they provide valuable insights into the delivery, effectiveness, and outcomes of primary care. This article offers a foundation for choosing suitable designs, addressing methodological challenges and contributing to evidence-based practice. As health services information systems develop and large datasets become more accessible, they offer valuable opportunities to design and conduct doctoral studies by posing relevant research questions within the secondary health data ecosystem.^25^ Future challenges include the development of data sharing policies and efforts to ensure critical aspects, such as architecture, metadata security and respecting privacy concerns. Longitudinal record linkage in sub-Saharan African countries^26,27^ holds great promise for expanding healthcare research capacity, including studies related to primary care and pressing regional questions.

### Primary versus secondary data

Building on these developments, a critical dimension concerns the data sources underlying observational studies. Historically, case–control studies and many cohort investigations relied on primary data collection, in which researchers directly recruited participants and gathered information on exposure, covariates and outcomes. Increasingly, however, entire studies can be conducted using secondary data sources, such as administrative health databases, electronic medical records or disease registries. This shift not only opens up opportunities but also introduces disparities: such infrastructures are well developed in some countries and health jurisdictions but remain limited or absent in many sub-Saharan African countries and other low- and middle-income settings.

For doctoral students in particular, this reality means that a project’s contribution may extend beyond the substantive health question or methodological innovation to include the navigation, curation and even development of robust data infrastructures. These efforts are not merely auxiliary but constitute significant incremental advances in research capacity, where secondary data sources are not fully established. In such contexts, researchers face a heightened responsibility to ensure transparency in data generation and to document related processes at the technical, system and governmental levels.

Although being ‘behind’ in data infrastructure may appear disadvantageous, it can also provide opportunities to shape new systems by explicitly incorporating data needs identified in doctoral projects – an opportunity often missed in more established research environments. Even seemingly simple elements, such as systematically recording why a primary care physician chose one treatment over another, can prove critical. Such information directly enhances the ability to address confounding bias and illustrates why propensity score-based analyses, when applied in the context of medical decision-making, can provide more reliable control for confounding than in settings where the determinants of exposure are less well understood.

### Classification- and prediction-oriented approaches

While this article has concentrated on traditional exposure-outcome effect estimation, observational data are increasingly utilised for classification and prediction purposes. Modern machine learning methods offer powerful tools for predicting health outcomes and stratifying risk at the individual level, often based on complex combinations of clinical, behavioural and contextual factors rather than a single exposure. Such personalised or stratified approaches may, in practice, be more intuitive and directly applicable than population-level averages. However, it is crucial to recognise that many prediction models, including those derived from machine learning, are inherently associational. They often encounter difficulties in transportability across different settings, which limits their usefulness for establishing underlying cause–effect relationships and for delivering actionable evidence to guide practice guidelines and policy decisions.

## Conclusion

Well-designed observational studies answer research questions that experimental designs may not be able to answer ethically or practically. Formulating a focused research question guided by appropriate frameworks and selecting a suitable study design are essential first steps in conducting rigorous observational studies. Mitigation of potential bias and confounding begins from design through to sampling and statistical analysis.

Increasingly, the concept of target trial emulation serves as a link to the randomised trial paradigm by framing observational studies as if they were RCTs, thereby improving causal interpretation while keeping real-world relevance. In this way, observational studies are not just alternatives when trials are unfeasible; they can be the preferred design choice, and in some cases, even better than RCTs, providing greater generalisability, the ability to explore ethically sensitive questions and insights that are directly relevant to healthcare delivery and policy.
